# 2025 FDA TIDES (Peptides and Oligonucleotides) Harvest

**DOI:** 10.3390/ph19020244

**Published:** 2026-01-30

**Authors:** Danah AlShaer, Othman Al Musaimi, Fernando Albericio, Beatriz G. de la Torre

**Affiliations:** 1 Department of Chemical Engineering, Imperial College London, London SW7 2AZ, UK; d.alshaer@imperial.ac.uk; 2 School of Pharmacy, Newcastle University, Newcastle upon Tyne NE1 7RU, UK; 3 Orthogonal Peptides Limited, London SW7 2AZ, UK; 4 School of Chemistry and Physics, University of KwaZulu-Natal, Durban 4001, South Africa; 5 Department of Inorganic and Organic Chemistry, University of Barcelona, 08028 Barcelona, Spain; 6 School of Laboratory Medicine and Medical Sciences, College of Health Sciences, University of KwaZulu-Natal, Durban 4001, South Africa; garciadelatorreb@ukzn.ac.za

**Keywords:** FDA approvals, drugs, peptides, oligonucleotides, fitusiran, donidalorsen, plozasiran, elamipretide, telisotuzumab vedotin-tllv

## Abstract

In 2025, the FDA approved 46 novel drugs, including four TIDEs (one peptide, three oligonucleotides, and one antibody drug conjugate containing peptide as a payload). The three approved oligonucleotide therapeutics—fitusiran, donidalorsen, and plozasiran—bring the total number of approved oligonucleotide drugs to 24 across 16 clinical indications since 1998. Fitusiran and donidalorsen are the first oligonucleotide therapies approved for antithrombin deficiency and hereditary angioedema, respectively, while plozasiran represents the second approved therapy for familial chylomicronemia syndrome. All three agents employ GalNAc-mediated hepatocyte targeting, highlighting the continued importance of liver-directed delivery platforms in oligonucleotide drug development and underscoring the growing clinical maturity of this therapeutic class. Peptide-based therapeutics continue to emerge as pioneering treatments for longstanding diseases. In 2025, elamipretide further expanded this paradigm by becoming the first disease-specific treatment approved for Barth syndrome. This review provides an overview of TIDES approved in 2025, with emphasis on their chemical structures, medical targets, modes of action, routes of administration, and associated adverse effects.

## 1. Introduction

**Figure 1:**
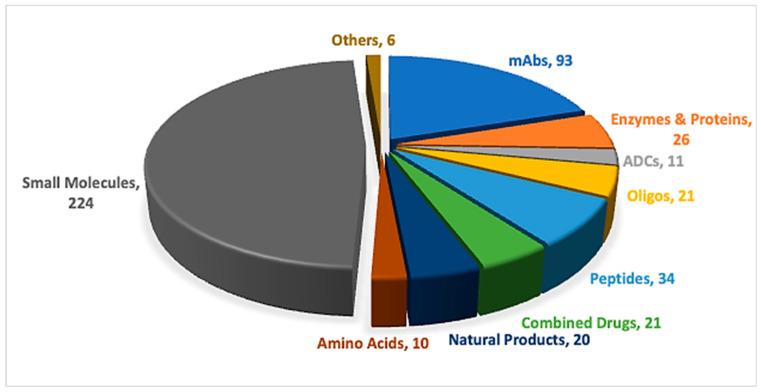
A total of 466 new drugs were approved by the Food and Drug Administration (FDA) between 2016 and 2025 [4,5]. ADCs, antibody–drug conjugates; mAbs, monoclonal antibodies; Oligos, oligonucleotides.

Peptides and oligonucleotides (TIDES) are increasingly competing within the pharmaceutical arena, driven by their broad range of applications across multiple therapeutic areas [1,2]. Advances in synthetic technologies have accelerated the development and regulatory approval of these important modalities [3]. In 2025, one peptide, three oligonucleotides, and one antibody–drug conjugate (ADC) incorporating a peptide payload were approved (Table 1) [4,5]. Overall, the number of newly approved new chemical entities (NCEs), 46, was slightly lower than that observed over the previous five years, yet remained higher than in 2022, during which only 37 NCEs were approved [6]. TIDES-based therapeutics continue to feature consistently among new approvals, underscoring their sustained role in addressing long-standing diseases through novel therapeutic strategies (Figure 1).

Table 1 summarizes the TIDES approvals in 2025, together with their indications, therapeutic targets, and routes of administration.

## 2. Oligonucleotides

This year concluded with the approval of three new oligonucleotide therapeutics, bringing the total number of FDA-approved oligonucleotide drugs to 24 since 1998, collectively addressing 16 distinct clinical indications (Table 2) [1,7]. Notably, two of the agents approved in 2025, fitusiran and donidalorsen, represent the first oligonucleotide therapeutics approved for their respective indications, antithrombin deficiency in hemophilia A or B [8] and hereditary angioedema (HAE) [9]. In contrast, plozasiran became the second oligonucleotide approved for the treatment of familial chylomicronemia syndrome (FCS) [10], together underscoring the expanding potential of oligonucleotides as highly targeted therapeutic modalities.

The 24 approved oligonucleotide therapeutics encompass a diverse range of mechanisms of action. These include aptamers developed for ocular indications such as macular degeneration and geographic atrophy associated with age-related macular degeneration (GA-AMD), as well as antisense oligonucleotides (ASOs) approved for nine disorders. Duchenne muscular dystrophy is a prominent example, with four FDA-approved ASOs enabling exon-skipping therapies targeting exons 51, 53, and 54. Following the approval of patisiran in 2018—the first small interfering RNA (siRNA) therapeutic—seven additional siRNA drugs have since received FDA approval, highlighting the growing significance of RNA interference–based therapies across a range of diseases [1,7].

Delivery strategies for oligonucleotide therapeutics have also evolved substantially. Early approaches included lipid nanoparticle–based delivery, as exemplified by patisiran in 2018. Subsequently, Alnylam introduced enhanced stabilized chemistry–*N*-acetylgalactosamine (ESC-GalNAc) conjugation with the approval of givosiran in 2019 [11], enabling targeted delivery to hepatocytes. This platform relies on conjugation of a GalNAc dendrimer to a chemically modified oligonucleotide to improve stability, potency, and liver-specific uptake via the asialoglycoprotein receptor. To date, nine approved oligonucleotide therapeutics have employed this GalNAc-based targeting strategy, all directed toward hepatocytes. More recently, the GalXC™ delivery platform was introduced with the approval of nedosiran in 2023 [12], in which GalNAc moieties are directly incorporated as appendages on modified nucleoside monomers within the oligonucleotide strand, achieving efficient hepatocyte targeting through an alternative structural design.

Among the 2025 approvals, the three oligos utilize ESC-GalNAc–mediated hepatocyte targeting. Conjugation of donidalorsen to a GalNAc ligand confers an approximately threefold enhancement in potency relative to its unconjugated oligonucleotide [9]. Although the GalNAc moieties are presented on distinct molecular scaffolds across these therapies, each achieves selective uptake by hepatocytes. 

### 2.1. Fitusiran (Qfitlia)

**Figure 2:**
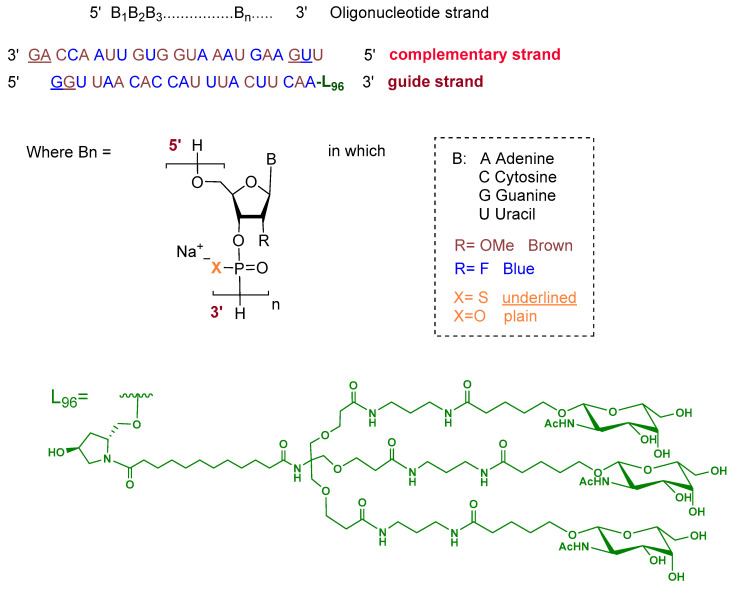
Chemical structure of fitusiran.

Fitusiran is a double-stranded siRNA formulated as its sodium salt. The guide strand is 21 nucleotides in length and is conjugated at the 3′ end to a triantennary *N*-acetylgalactosamine (GalNAc) dendrimer ligand (L96) to enable targeted uptake by hepatocytes. The ribose units of the guide strand are chemically modified with alternating 2′-fluoro and 2′-O-methoxy groups. All nucleotides are linked via phosphodiester bonds, except for two phosphorothioate linkages at the 5′ terminus. The complementary (passenger) strand consists of 23 nucleotides with similar ribose modifications and contains two phosphorothioate linkages at both termini, with the remaining linkages being standard phosphodiesters (Figure 2) [8].

Fitusiran is an antithrombin-directed small interfering RNA approved for routine prophylaxis to reduce bleeding frequency in adults and pediatric patients aged 12 years and older with hemophilia A or B, with or without factor VIII or IX inhibitors [8].

Hemophilia is an X-linked inherited bleeding disorder caused by mutations in the *F8* or *F9* genes. These mutations result in deficiencies of clotting factors VIII or IX, respectively. The lack of these factors impairs thrombin generation. Thrombin is a central serine protease that converts fibrinogen into fibrin, which is essential for clot formation. As a result, patients are more susceptible to spontaneous and trauma-related bleeding, including bleeding into joints and the brain [13,14,15]. Antithrombin (AT) is a serine protease inhibitor that negatively regulates coagulation by inactivating thrombin and other key clotting enzymes [13,14,15]. Fitusiran reduces plasma AT levels by inducing RNA interference–mediated degradation of AT messenger RNA in hepatocytes. This restores thrombin generation and is associated with lower bleeding rates [8,15].

Fitusiran exhibits dose-proportional pharmacokinetics following subcutaneous administration, with peak plasma concentrations (Cmax) reached within 2.9–3.8 h (Tmax) and a half-life of approximately 5.6–8.0 h, depending on the dose. The drug demonstrates high protein binding (~96.6%) and a large volume of distribution (431–570 L at steady state) with no accumulation observed after monthly dosing. Fitusiran is metabolized by endo- and exo-nucleases to progressively shorter oligonucleotides and is not a substrate for CYP450 enzymes or transporters. Approximately 15% of a 50 mg dose is excreted unchanged in urine, and apparent clearance ranges from 42 to 51 L/h [8,15].

Qfitlia is administered via subcutaneous injection. The most frequently reported adverse reactions (incidence > 10%) include viral infections, nasopharyngitis, and bacterial infections [8]. It was developed by the Genzyme corporation and approved by the FDA on 28 March 2025 [16].

### 2.2. Donidalorsen (Dawnzera^TM^)

**Figure 3:**
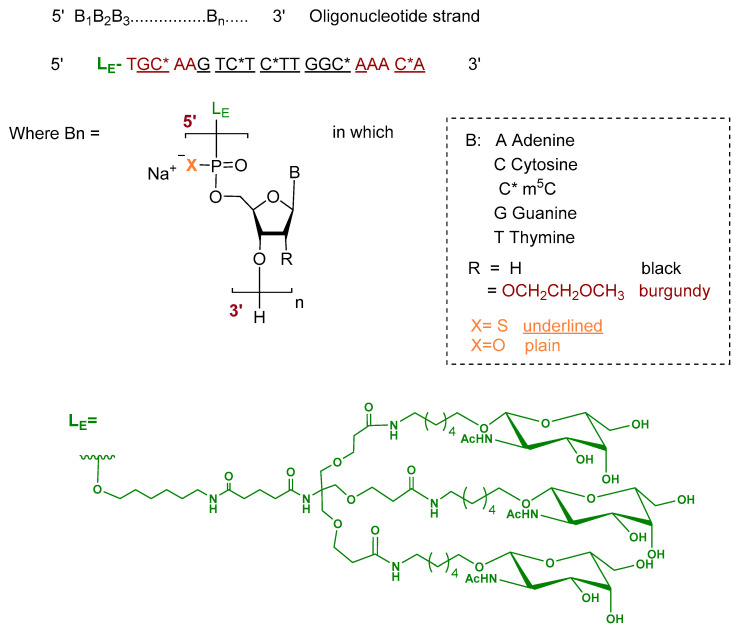
Chemical structure of donidalorsen.

Donidalorsen is an ASO formulated as a sodium salt and consists of a 20-nucleotide sequence. The five nucleotides at each terminus contain 2′-O-methoxyethyl—modified ribose units, while the central ten nucleotides are DNA monomers. The nucleotides are predominantly linked by phosphorothioate inter-nucleotide linkages, with the remaining linkages comprising phosphodiester bonds. Selected cytidine residues incorporate 5-methylcytosine as the nucleobase. The ASO is conjugated at the 5′ terminus to a GalNAc dendrimer, which facilitates targeted uptake into hepatocytes (Figure 3) [9,17].

Donidalorsen is the first-of-its-kind prekallikrein-directed ASO indicated for prophylaxis to prevent HAE attacks in adult and pediatric patients aged 12 years and older [9,17].

HAE is a rare genetic disorder characterized by recurrent, potentially life-threatening episodes of tissue swelling affecting multiple organs. It typically manifests in childhood and is caused by mutations in the SERPING1 gene, leading to deficient or dysfunctional C1 esterase inhibitor (C1-INH). Loss of C1-INH results in dysregulation of the plasma contact system, with uncontrolled activation of plasma kallikrein and factor XII, leading to increased vascular permeability and angioedema. In HAE, plasma prekallikrein (PKK) is overproduced. Donidalorsen targets PKK mRNA, inducing RNase H1–mediated degradation, thereby reducing PKK protein synthesis and lowering the risk of angioedema attacks [18].

In a phase 3 clinical study involving adult and pediatric patients (≥12 years) with HAE types 1 or 2, Dawnzera 80 mg reduced plasma PKK concentrations by Week 24, achieving a mean reduction of 73% with every-4-week dosing and by 47% with every-8-week dosing, compared with a 2% increase in the placebo group [9,17].

Following subcutaneous administration, donidalorsen reached a median time to peak concentration of approximately 2 h. It distributes predominantly to the liver and kidney cortex and demonstrates high plasma protein binding (>98%). The terminal elimination half-life is ~1 month, with an initial distribution half-life of ~5 h. The ASO is metabolized by nucleases and is not a CYP substrate, while the linker undergoes hydrolytic and oxidative metabolism. Renal elimination is minimal, with <1% of unchanged drug excreted in urine [9,17].

Dawnzera is administered subcutaneously; the most common adverse events include injection-site reactions, upper respiratory tract infection, urinary tract infection, and abdominal discomfort [9]. It was developed by Ionis pharmaceuticals and was approved by FDA on 21 August 2025 [19].

### 2.3. Plozasiran (Redemplo)

**Figure 4:**
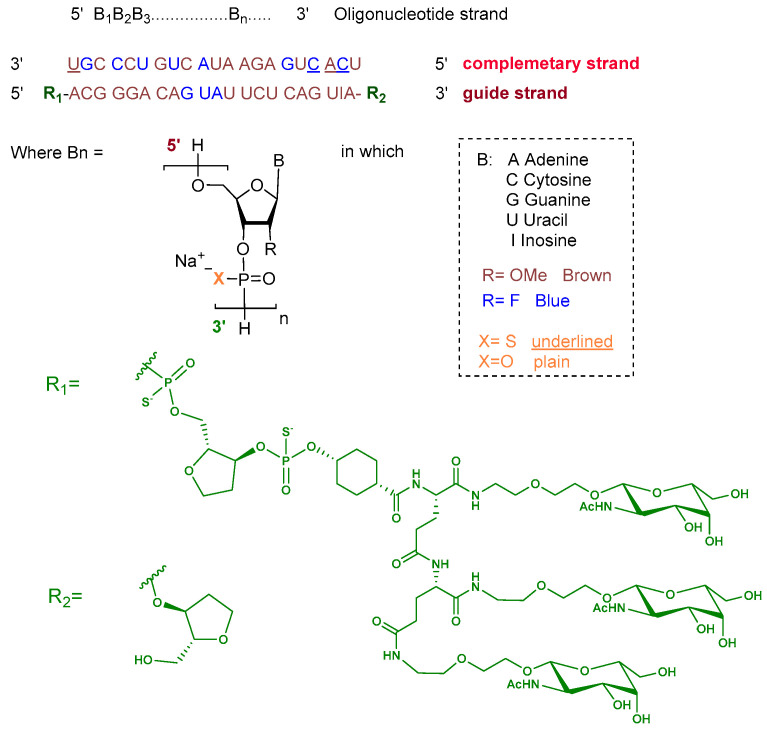
Chemical structure of plozasiran.

Plozasiran is an siRNA consisting of the sodium salt of two complementary strands, each 21 nucleotides in length. The ribose units are chemically modified with either 2′-fluoro or 2′-O-methoxy substituents to enhance stability. Nucleotides within the guide strand are connected via standard phosphodiester linkages, and the strand is conjugated at its 5′ terminus to a triantennary GalNAc dendrimer to facilitate hepatocyte targeting. The complementary strand contains phosphorothioate linkages at both termini to improve nuclease resistance (Figure 4) [10].

Plozasiran is indicated as an adjunct to dietary management for the reduction in triglyceride levels in adult patients with familial chylomicronemia syndrome (FCS) [10].

Familial chylomicronemia syndrome (FCS) is a rare metabolic disorder caused by mutations in the lipoprotein lipase (LPL) gene, resulting in a dysfunctional enzyme incapable of mediating triglyceride hydrolysis. Under physiological conditions, LPL is expressed on the luminal surface of vascular endothelial cells, where it catalyzes the lipolysis of circulating triglycerides contained in chylomicrons and other triglyceride-rich lipoproteins (TGRLs), typically within 3–4 h following a meal. Loss of functional LPL activity leads to impaired clearance of chylomicrons, causing their persistence in the circulation even during fasting. This results in severe hypertriglyceridemia, with subsequent accumulation of chylomicrons in the bloodstream, which can impair microvascular blood flow and lead to organ damage, particularly in the pancreas and liver, or deposition in peripheral tissues such as skin and retina [20].

In FCS, apolipoprotein C-III (APOC3) worsens hypertriglyceridemia by inhibiting both LPL–mediated and LPL-independent clearance of chylomicrons. Plozasiran targets the mRNA encoding APOC3, triggering its catalytic degradation and thereby suppressing APOC3 translation and protein expression. The reduction in APOC3 levels alleviates its inhibitory effect on LPL activity and enhances LPL-independent hepatic clearance of triglyceride-rich lipoproteins, leading to an overall improvement in disease manifestations [21].

Following subcutaneous administration across a dose range of 10–100 mg, plozasiran attains a peak plasma concentration (Cmax) of 68.5 ng/mL at a median Tmax of approximately 6 h. After repeated subcutaneous dosing at 25 mg, the apparent volume of distribution is approximately 146 L, consistent with distribution mainly within plasma and extracellular fluid prior to uptake by hepatocytes for suppression of APOC3 mRNA expression. In vitro, plozasiran exhibits moderate plasma protein binding (~78%). The compound is primarily metabolized by nucleases to shorter oligonucleotide fragments, with renal excretion accounting for approximately 16–19% of the administered dose. Plozasiran displays a terminal plasma elimination half-life of 3–4 h and a mean apparent systemic clearance of 33.8 L/h [10].

Redemplo is administered subcutaneously. The most common adverse reactions observed in Redemplo-treated patients (incidence ≥10% and occurring at least 5% more frequently than with placebo) include hyperglycemia, headache, nausea, and injection-site reactions [10].

Redemplo was developed by Arrowhead Pharmaceuticals and received FDA approval on 18 November 2025 [22].

## 3. Peptides

Peptides have established a strong presence in the pharmaceutical landscape, with a total of 34 FDA approvals between 2016 and 2024 [7]. They span a wide range of therapeutic applications, including anticancer, antimicrobial, and neurological disorders, and are integral components of advanced modalities such as ADCs and peptide–drug conjugates (PDCs). Peptides have also demonstrated significant clinical relevance in cardiovascular disease, with previously FDA-approved examples including eptifibatide (Integrilin), bivalirudin (Angiomax), nesiritide (Natrecor), and icatibant (Firazyr) [2]. In 2025, this trajectory continued with the FDA approval of elamipretide (Forzinity) for improving muscle strength in adult and pediatric patients with Barth syndrome (≥30 kg), and telisotuzumab vedotin-tllv (Emrelis), an ADC containing a peptide payload, for the treatment of previously treated, locally advanced or metastatic non-squamous non-small cell lung cancer with high c-Met protein overexpression.

### 3.1. Elamipretide (Forzinity)

**Figure 5:**
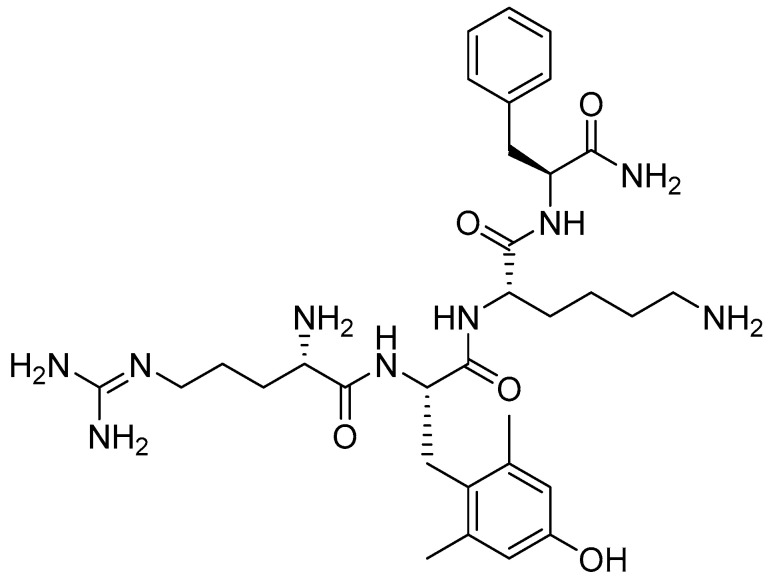
Chemical structure of elamipretide.

Elamipretide is a synthetic tetrapeptide containing one unusual amino acid residue, namely (S)-2-amino-3-(4-hydroxy-2,6-dimethylphenyl)propanoic acid (Figure 5) [23].

Elamipretide is indicated for improving muscle strength in adult and pediatric patients with Barth syndrome weighing at least 30 kg [23,24]. It is a novel mitochondrial-targeted agent that enhances mitochondrial energy production by selectively binding cardiolipin within the inner mitochondrial membrane, thereby improving mitochondrial morphology and function. Elamipretide became the first disease-specific therapy approved for the treatment of Barth syndrome, an ultra-rare X-linked recessive genetic disorder. In addition, elamipretide is currently under phase III clinical development for the treatment of dry age-related macular degeneration and mitochondrial myopathies [25].

In the elamipretide clinical program, 12 male patients (12–35 years) with genetically confirmed Barth syndrome received daily subcutaneous elamipretide (40 mg) [23]. The study employed a randomized, double-blind, placebo-controlled crossover design with two 12-week treatment periods separated by a 4-week washout [23]. Ten patients completed the randomized phase and entered an open-label extension, with treatment durations of up to 168 weeks (192 weeks in three patients) [23].

Clinical evaluation has shown that elamipretide is generally well tolerated, with most adverse events being mild to moderate in severity. However, the study by Karaa and colleagues provides Class I evidence that elamipretide does not significantly improve the 6 min walk test (6MWT) performance or fatigue outcomes at 24 weeks compared with placebo in patients with primary mitochondrial myopathy [26]. Furthermore, Allingham et al. found that elamipretide was generally safe and well tolerated in patients with intermediate age-related macular degeneration (AMD) and high-risk drusen (HRD). Exploratory analyses suggested a beneficial effect on visual function, particularly under low-luminance conditions [27].

Elamipretide exhibits dose-proportional exposure over a range of 2–80 mg following daily subcutaneous administration, with minimal accumulation [23]. Peak plasma concentrations are achieved within 0.5–1 h, and absolute bioavailability is approximately 92%, with comparable exposure following thigh or abdominal injection [23]. The drug distributes throughout total body water, with a volume of distribution of ~0.5 L/kg and low plasma protein binding (~39%). Elamipretide is metabolized via sequential *C*-terminal degradation to inactive M1 and M2 metabolites and is primarily excreted in urine, with nearly complete recovery of the administered dose within 48 h in patients with normal renal function [23]. Elamipretide exposure (AUC) increased with worsening renal function, rising by 39% in patients with mild renal impairment (creatinine clearance 60–89 mL/min), 75% with moderate impairment (30–59 mL/min), and 125% with severe impairment (<30 mL/min, not on dialysis), as defined by 24 h measured urinary creatinine clearance [23]. Despite increased exposure, minimal accumulation was observed with daily dosing across all degrees of renal impairment. Exposure to the inactive M1 (tripeptide) and M2 (dipeptide) metabolites increased substantially in patients with severe renal impairment, by up to 280% and 640%, respectively [23]. Pharmacokinetic analyses based on estimated glomerular filtration rate (CKD-EPI) were consistent with these findings and support current dosing recommendations. In contrast, hepatic impairment is not expected to affect elamipretide pharmacokinetics, as no hepatic metabolism was observed in vitro [23].

The drug is administered via subcutaneous injection, with injection-site reactions reported as the most common adverse effect, including erythema, pain, induration, pruritus, bruising, and urticaria [23]. It has been developed by Stealth BioTherapeutics and approved by the FDA on 19 September 2025 [24].

### 3.2. Telisotuzumab Vedotin-Tllv (Emrelis)

**Figure 6:**
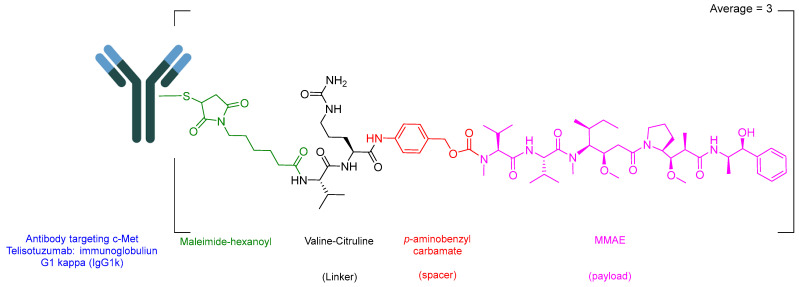
Chemical structure of Emrelis.

Telisotuzumab vedotin-tllv (Emrelis) is a c-Met–directed antibody–drug conjugate (ADC). Emrelis consists of a humanized immunoglobulin G1 kappa (IgG1κ) monoclonal antibody targeting c-Met, the hepatocyte growth factor receptor, conjugated via a protease-cleavable Val-Cit linker to monomethyl auristatin E (MMAE), a potent microtubule-disrupting agent (Figure 6) [28]. The monoclonal antibody component is produced in a mammalian expression system using Chinese hamster ovary (CHO) cells, while the drug–linker moiety is obtained through chemical synthesis. Each antibody molecule is conjugated to an average of approximately three monomethyl auristatin E (MMAE) molecules [29].

Emrelis is used for the treatment of previously treated, locally advanced or metastatic non-squamous non-small cell lung cancer (NSCLC) with high c-Met protein overexpression [29]. Upon binding to c-Met–expressing tumor cells, telisotuzumab vedotin-tllv is internalized, followed by intracellular cleavage and release of MMAE. The released payload disrupts the microtubule network in actively dividing cells, leading to cell cycle arrest and apoptotic cell death. Telisotuzumab vedotin-tllv has demonstrated significant antitumor activity in NSCLC xenograft models.

The safety profile of Emrelis is based on 168 patients with locally advanced or metastatic EGFR wild-type non-squamous NSCLC with c-Met overexpression treated with 1.9 mg/kg IV every 2 weeks in the LUMINOSITY study. The median age was 64.5 years, 70% were male, and the majority were White (65%) or Asian (33%). Patients were exposed for ≥6 months (42%) and >1 year (11%) [29]. Serious adverse reactions occurred in 35% of patients, most commonly ILD/pneumonitis (5%), pneumonia (5%), peripheral neuropathy (3.6%), and pleural effusion (2.4%), with fatal reactions reported in 5% [29]. Permanent discontinuations due to adverse events occurred in 30%, and dose interruptions and reductions occurred in 44% and 28% of patients, respectively [29].

Plasma exposure of both the ADC and unconjugated MMAE increased proportionally over the dose range of 1.2–3.3 mg/kg [29]. Peak ADC concentrations occurred at the end of intravenous infusion, whereas unconjugated MMAE reached a median maximum concentration (Cmax) of 29 µg/mL (CV 43%) approximately 5 days after dosing. The AUC_0_–τ (0–14 days) was 2130 µg·h/mL (CV 55%) for the ADC and 405 ng·h/mL (CV 64%) for unconjugated MMAE, with minimal accumulation observed for either component [29]. The estimated volume of distribution of telisotuzumab vedotin-tllv is 3.4 L, and MMAE exhibits plasma protein binding of 68–82% in vitro [29]. The elimination half-life is approximately 3 days for the ADC and 4 days for MMAE, with estimated clearances of 1.3 L/day and 76 L/day, respectively [29]. Telisotuzumab vedotin-tllv is expected to undergo catabolism to peptides, amino acids, and unconjugated MMAE, while MMAE is primarily metabolized by CYP3A4 [29]. No clinically meaningful pharmacokinetic differences were observed based on age, sex, race, body weight, mild to moderate renal impairment, or mild hepatic impairment [29]. The effects of severe renal impairment, end-stage renal disease, or moderate to severe hepatic impairment remain unknown [29].

The drug is administered intravenously. The most common adverse reactions (≥20%) include peripheral neuropathy, fatigue, decreased appetite, and peripheral edema. Frequently observed grade 3 or 4 laboratory abnormalities (≥2%) include decreased lymphocyte counts, decreased hemoglobin, phosphorus, sodium, and calcium levels, as well as increased glucose, alanine aminotransferase, and gamma-glutamyl transferase levels [29]. Emrelis was developed by AbbVie Inc. North Chicago, Illinois, USA, and received the FDA approval on 14 May 2025 [28,30].

## 4. Conclusions

TIDES continue to emerge not only as conventional therapeutics but also as innovative solutions for diseases that are difficult to treat using traditional approaches. Their versatility and precision place them in a distinguished class alongside established therapeutic modalities such as small molecules and biologics. Hundreds of peptide-based candidates are currently in the development pipeline for a wide range of diseases and applications [31], including therapeutic, diagnostic, and theranostic uses [32]. These candidates encompass diverse modalities such as peptide-based inhibitors [33], peptide–drug conjugates (PDCs) [32], and other emerging peptide-based platforms. The approval of five TIDES-based therapeutics in 2025 further underscores their growing clinical relevance and highlights their expanding role in the development of effective treatments for complex and previously unmet medical needs.

The approval of 24 oligonucleotide therapeutics over the past two and a half decades illustrates the maturation of this drug class from experimental molecules to clinically validated therapies. Beyond expanding the range of treatable indications, recent approvals highlight how innovation in chemical design and delivery platforms, such as GalNAc conjugation and GalXC™, has enabled precise targeting, improved potency, and enhanced therapeutic outcomes. These advances not only demonstrate the versatility of oligonucleotides across diverse mechanisms of action but also underscore their potential to address previously unmet medical needs. Looking forward, continued refinement of delivery strategies and molecular engineering is likely to drive further clinical adoption, positioning oligonucleotide therapeutics as a central component of precision medicine.

The approvals of elamipretide and Emrelis highlight the growing impact of targeted and mechanism-driven therapeutics in addressing unmet medical needs. Elamipretide represents a milestone as the first disease-specific treatment for Barth syndrome, offering a novel therapeutic option for patients affected by this ultra-rare X-linked recessive disorder. Similarly, Emrelis marks a significant advancement in oncology as the first and only approved therapy for non-squamous NSCLC with high c-Met protein overexpression. Its demonstrated clinical efficacy, combined with targeted delivery of a potent cytotoxic payload, underscores the advantages of precision medicine by achieving effective tumor control while minimizing off-target toxicity. Together, these approvals exemplify the continued evolution of peptide-based and ADC therapies in expanding the therapeutic landscape for rare and complex diseases.

## Figures and Tables

**Table 1:** Summary of FDA-approved TIDES in 2025.

#	Active Ingredient (Trade Name)	Indication	Therapeutic Target	Administration Route
Oligonucleotides				
1	Fitusiran (Qfitlia)	To prevent or reduce the frequency of bleeding episodes in adult and pediatric patients aged 12 years and older with hemophilia A or B with or without factor VIII or IX inhibitors.	Antithrombin-III mRNA	Subcutaneous
2	Donidalorsen (Dawnzera)	Indicated for prophylaxis to prevent attacks of hereditary angioedema (HAE) in adult and pediatric patients 12 years of age and older	PKK mRNA	Subcutaneous
3	Plozasiran (Redemplo)	Indicated as an adjunct to diet to reduce triglycerides in adults with familial chylomicronemia syndrome (FCS).	Apoc-III mRNA	Subcutaneous
Peptides				
4	Elamipretide (Forzinity)	Improving muscle strength in adult and pediatric patients with Barth syndrome weighing at least 30 kg	Mitochondria	Subcutaneous
ADC				
5	Telisotuzumab vedotin-tllv (Emrelis)	Treatment of previously treated locally advanced or metastatic non-squamous non-small cell lung cancer (NSCLC)	c-Met	Intravenous

PKK: prekallikrein; APOC-III, apolipoprotein C-III.

**Table 2:** Overview of the 24 FDA-approved oligonucleotide therapeutics (1998–2025), highlighting the evolution of clinical indications, modes of action, and delivery technologies.

	1998	2004	2013	2016	2018	2019	2020	2021	2022	2023	2024	2025
Cytomegalovirus retinitis	Fomivirsen											
Macular degeneration		Pegaptanib										
Homozygous familial hypercholesterolemia			Mipomersen									
DMD (Exon 51, 53, 54)				Eteplirsen		Golodirsen	Viltolarsen	Casimersen				
Hepatic veno-occlusive disease				*Defibrotide*								
Spinal muscular atrophy				Nusinersen								
Hereditary Transthyretin Amyloidosis (haTTR)					Patisiran				Vutrisiran	Eplontersen		
					Inotersen							
Hepatic porphyria						Givosiran						
Primary hyperoxaluria type 1							Lumasiran			Nedosiran		
Hypercholesterolemia								Inclisiran				
Geographic atrophy (GA-AMD)										Avacincaptad pegol		
Amyotrophic lateral sclerosis										Tofersen		
Myelodysplastic syndromes (MDS)											Imetelstat	
Familial chylomicronemia syndrome (FCS)											Olezarsen	Plozasiran
Hereditary angioedema (HAE)												Donidalorsen
Hemophilia A or B												Fitusiran

Mode of action: Anstisense oligonucleotide (ASO), Apatmer, small interfering RNA (siRNA), *not elucidated*. Fileds’ colors show the drug delivery technology used; yellow, liposome; green, ESC-NGalNAc; pink, GalXC™.

## Data Availability

No new data were created or analyzed in this study. Data sharing is not applicable to this article.
